# Rapidly Expanding Pediatric Post Radiation Brainstem Cavernoma Presenting with Singultus

**DOI:** 10.7759/cureus.4157

**Published:** 2019-02-28

**Authors:** Troy Dawley, Gary Rajah, William Kupsky, Abilash Haridas

**Affiliations:** 1 Neurosurgery, St. John Providence Hospital, Southfield, USA; 2 Neurosurgery, Wayne State University School of Medicine, Detroit, USA; 3 Pathology, Wayne State University School of Medicine, Detroit, USA

**Keywords:** post radiation, cavernoma, singultus, pnet

## Abstract

Here we present a pediatric patient status post resection of a primitive neuroectodermal tumor (PNET) with cranial/spinal radiation and development of a medullary cavernoma seven years after radiation therapy. The patient’s cavernoma demonstrated rapid symptomatic growth in six weeks resulting in the presentation of intractable hiccups (singultus). The patient underwent resection of the cavernoma with cessation of the hiccups. We also review the pathology and possible mechanisms of such rapid growth of this post-radiation cavernoma as well as advise surveillance for patients with such lesions, as their course may be different from that of sporadic cavernomas.

## Introduction

There is a well-described link between early radiation therapy in children (prior to 10 years) and future cavernoma development with a median time to development of post radiation cavernoma of ten and a half years (<3000 cGy dose), and about five years (>3000 cGy) [1]. Medullary cavernomas have been previously reported to be associated with singultus [2]. Here we describe a pediatric patient with an intramedullary cavernoma presenting with singultus, and discuss why post-radiation brainstem lesions may follow a natural history different from that of incidental brainstem cavernous malformations. Informed consent was obtained from all individual participants included in the study.

## Case presentation

**Figure 1:**
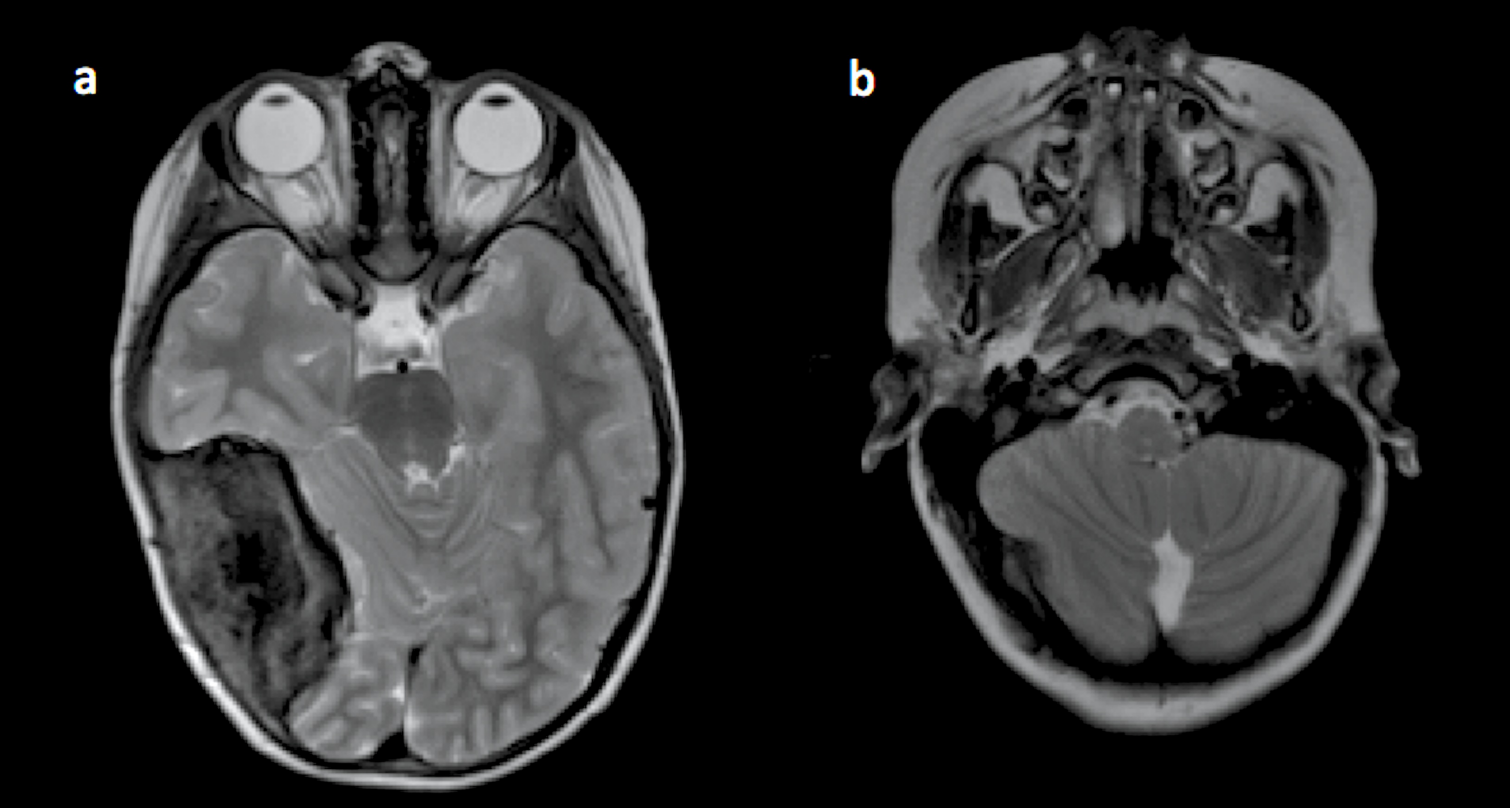
Presenting magnetic resonance imaging (MRI) prior to cavernoma formation (a) T2 axial MRI demonstrating a large R parietal lesion consistent with melanotic primitive neuroectodermal tumor (PNET), which did undergo gross total resection. (b) T2 axial MRI from 2007 demonstrating no lesions in the right medullary region.

A 14-year-old male presented with medically refractory hiccups and vomitus with a history of a post-radiation medullary cavernoma that acutely enlarged with significant surrounding edema. Originally, he presented at five years of age after a fall, and was incidentally found to have a right temporo-parietal and posterior fossa melanotic primitive neuroectodermal tumor (PNET, Figure 1). He underwent gross total resection and was treated with adjuvant chemotherapy and radiation.

**Figure 2:**
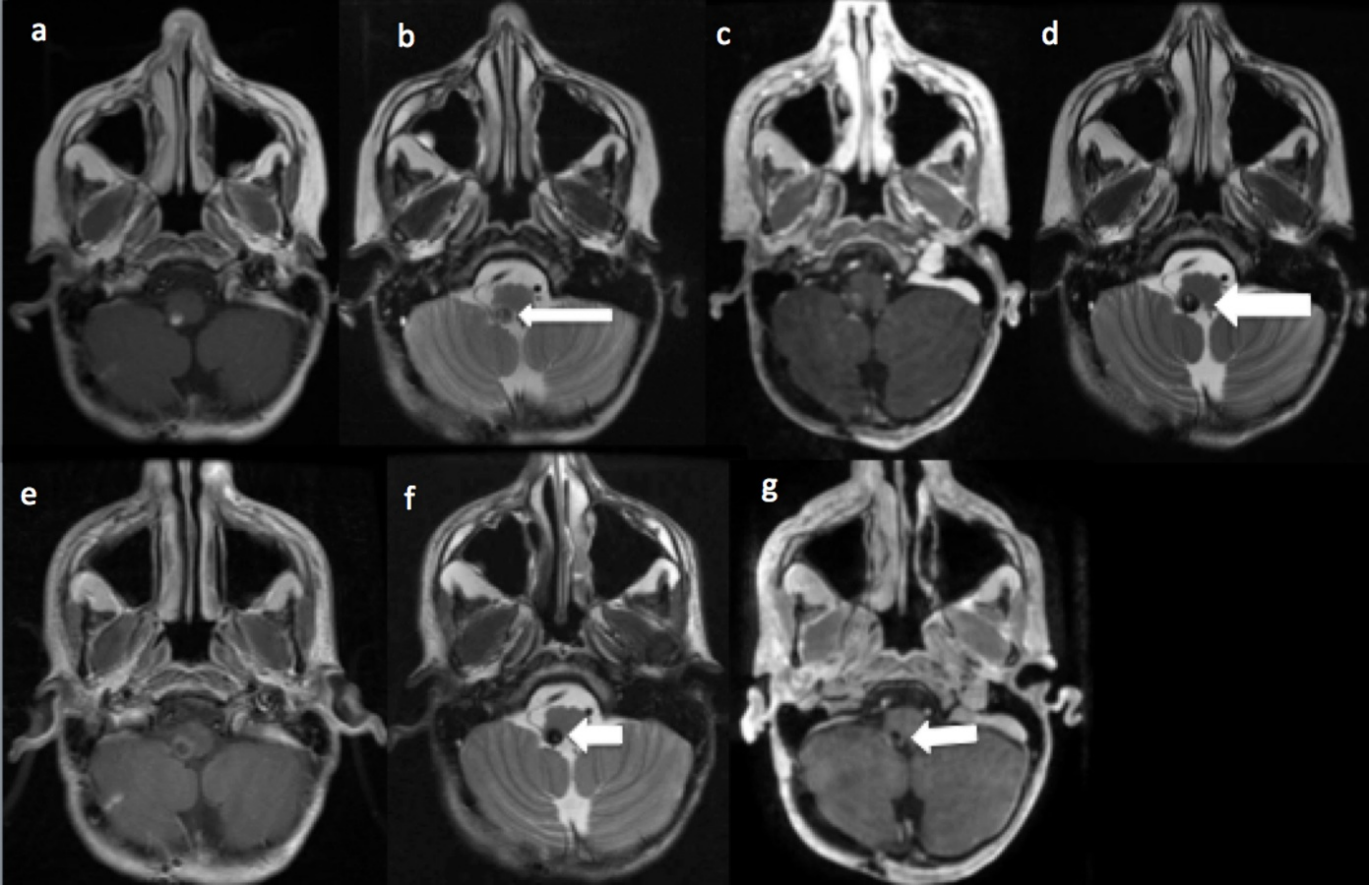
Right medullary cavernoma initially diagnosed on routine follow-up imaging seven years after the initial resection of the primitive neuroectodermal tumor (PNET) Right medullary cavernoma size over time. (a/b): Axial T1 and T2 images depicting first radiographic evidence of the right medullary cavernoma (white arrow), with some T1 with contrast enhancement seven years after initial surgery/radiation for melanotic PNET. (c/d): Axial T1 and T2 images depicting one-year follow-up study after initial cavernoma findings demonstrating less T1 with contrast enhancement, and small increase in size on T2 image (white arrow). (e/f): T1 and T2 images depicting cavernoma six weeks prior to presentation with intractable hiccups. The pattern of enhancement on T1 image is now ring like, but the overall size on T2 image is slightly smaller. (g): Post-resection T1 with contrast revealing complete resection of previous cavernoma (white arrow).

The amount of radiation received was 3600 cGy to the entire neuroaxis with a 5580 cGy boost to the tumor field. Approximately seven years after radiation, he presented with intermittent hiccups for two weeks. A brain magnetic resonance imaging (MRI) revealed a 4 mm medullary cavernoma that had minimal mass effect or edema present (Figure 2). The hiccups were managed medically for over three years. This included an extensive gastrointestinal workup, thoracic bracing, and behavioral modifications, and several medications.

**Figure 3:**
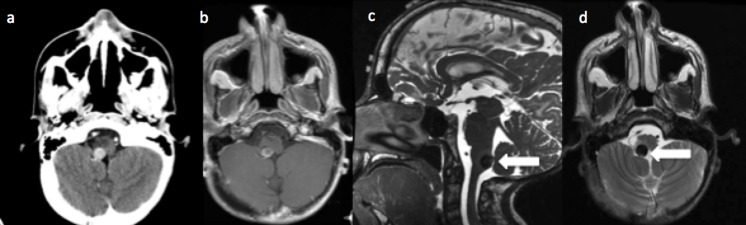
Presenting magnetic resonance imaging (MRI) after worsening hiccups (a) Computed tomography (CT) with contrast showing 1 cm enhancing lesion in R posterior medulla (40% increase in size from six weeks prior). (b) T1 with contrast axial MRI showing enhancing portion of cavernoma. (c) T2 high-resolution sagittal MRI depicting cavernoma (white arrow) location in lower posterior, right medulla. (d) T2 axial MRI showing the cavernoma (white arrow) with surrounding brainstem edema.

At the age of 14, he then presented to our emergency department with singultus and vomiting for three days. His singultus was refractory to medical management and hindered his ability for oral intake. This disrupted his normal breathing synchrony as well as sleep pattern. Repeat MRI imaging (Figure 3) showed that the cavernoma had acutely enlarged from 6 mm to 10 mm over a six-week period with significant surrounding edema. The persistent hiccups and radiological growth prompted surgical intervention. He underwent a midline suboccipital craniotomy and partial C1 laminectomy.

The lesion was approached using a right lazy hockey stick durotomy and a subtonsillar approach. Arachnoid dissection of the right tonsil allowed elevation off the medulla. Pial representation was seen on the lateral wall of the obex and the right lower mid-medulla. This corridor was opened sharply and circumferential dissection of the cavernoma was performed with motor and somatosensory evoked potential monitoring. The surrounding hemosiderin-stained tissue was left in place. Please refer to Video 1.

**Video 1:** Surgical video

**Figure 4:**
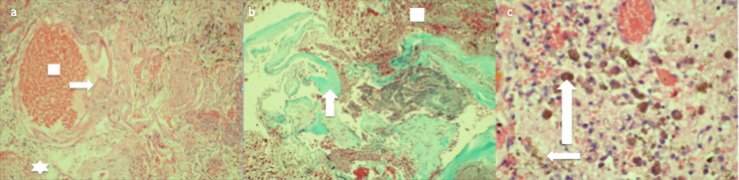
Histopathological examination (a) Hematoxylin and eosin stain from resected medullary cavernoma displaying large single layered endothelium-lined channels (arrow) with red blood cells within (box). Small amounts of intervening glial tissue are present suggesting the growing nature of the lesion (star). (b) Gomori trichrome stain with collagen stained green showing large sclerotic vascular channels (arrow). Surrounding immature granulation tissue suggests a growing nature of the lesion (square). (c) High power hematoxylin and eosin field revealing granulation tissue (small arrow), with abundant hemosiderin-laden macrophages (long arrow).

Histopathological examination revealed single layer endothelium-lined channels with intervening glial tissue and immature granulation tissue displaying the growing nature of this lesion (Figure 4). Post-operative imaging revealed total resection of the cavernoma and his hiccups ceased immediately. The post-operative course was routine and he was discharged home in two days.

## Discussion

Prior brain radiation has several long-term effects including secondary tumors and cavernomas. One large series found a 3.4% incidence of cavernomas following radiotherapy for brain tumors [3]. Brainstem cavernomas can present with a variety of symptoms, rarely including singultus. Spontaneous and familial medullary cavernomas resulting in singultus, while not common, have also been described with one review by Lee et al. reporting six patients with a mean age of 34 years (26-40) undergoing surgery [2]. This review found an incidence of hiccups ranging from 3 to 27% with medullary lesions, lesion size ranged from under 1 cm to 2.2 cm and hiccup duration ranged from 15 days to three years, with all patients having a sudden onset. All hiccups resolved post operatively in this review.

Surgery is the treatment of choice for symptomatic brainstem cavernomas coming to the pial surface. A large series of 100 patients by Porter et al. [4] reported 87% of patients undergoing resection were the same or better. Permanent morbidity occurred in 12%. Surgical approaches to the brainstem are numerous, and in a study of 300 cases, the most common approach is the suboccipital craniotomy with or without the telovelar approach. Brown et al. have described the selection of a surgical approach as a “two-point method” where the first point is in the middle of the lesion and the next is the area where it comes to/nearest to the pial surface. An extension of this line is the approach to the lesion [5]. Radiation therapy has been reported for deep seated lesions, however, its efficacy is debated given a two-year latency period where the hemorrhage rate in one study was 8.8% per year [6].

The mechanism of medullary cavernoma and hiccups is debated, but many theories exist including (1) disruption of the GABA inhibitory control via the raphe magnus nucleus of the hiccup arc [7], (2) irritation of the medullary reticular activating system, near nucleus ambiguus and the obex [8], and lastly (3) involvement of Mollaret’s myoclonic triangle and the olive [2]. Our surgical approach was planned with regards to this anatomy. The cavernoma described herein came to the surface at the obex and more posteriorly in the region of the inferior cerebellar peduncle, dorsal vagal motor nuclei, and nucleus tractus solitarius. We decided to avoid entering though the obex to avoid further disruption at the area postrema and leave the ventricular surface intact. The location allowed for access via the cerebellomedullary fissure, and pial entry just below the inferior loop of the tonsillomedullary portion of the right posterior inferior cerebellar artery (PICA).

A large literature review recommends only offering surgery to patients who have at least one episode of hemorrhage and have pial representation [9]. Many factors that have been determined to help predict outcomes include large lesion size, crossing midline, older age, presence of developmental venous anomaly, and a larger time interval from hemorrhage to surgical intervention [10].

## Conclusions

This report has detailed a rare rapidly expanding post radiation medullary cavernoma in a pediatric patient resulting in singultus. This can be extremely distressing and debilitating and prompt treatment is necessary. Although surgery in the brainstem is technically challenging, it remains a safe and viable option for symptomatic cavernomas that come to the pial surface.
